# Ovarian dysgerminoma with a metastasis to para‐aortic lymph node

**DOI:** 10.1002/ccr3.5227

**Published:** 2021-12-13

**Authors:** Rieko Kawase, Shunji Suzuki

**Affiliations:** ^1^ Department of Obstetrics and Gynecology Nippon Medical School Tokyo Japan

**Keywords:** complete surgical staging, dysgerminoma of ovary, metastasis of para‐aortic lymph node

## Abstract

We present a case of dysgerminoma of the right adnexa with an infiltration to the right wall of the uterus and a metastasis of para‐aortic lymph node.

## Graphical abstract

A complete surgical staging may be usually needed in cases of ovarian dysgerminoma. Fertility‐sparing surgery will be allowed only for young women with limited tumor spread as results of detailed examination.

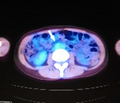

A 49‐year‐old nulliparous woman visited our hospital due to abdominal fullness. Her general condition was good. Magnetic resonance imaging revealed a 10.5 × 8.5 × 6.7 cm solid, enhancing right ovarian mass (Figure 1). Positron‐emission tomography and computed tomography (PET/CT) showed swollen lymph nodes in the abdominal para‐aorta (Figure 2). A complete surgical staging including para‐aortic lymphadenectomy for ovarian cancers was performed. The pathological results showed dysgerminoma of the right adnexa with an infiltration to the uterine wall and a metastasis of para‐aortic lymph node (PAN) (FIGO [International Federation of Gynecology and Obstetrics] ⅢC, Figure 3).

**FIGURE 1:**
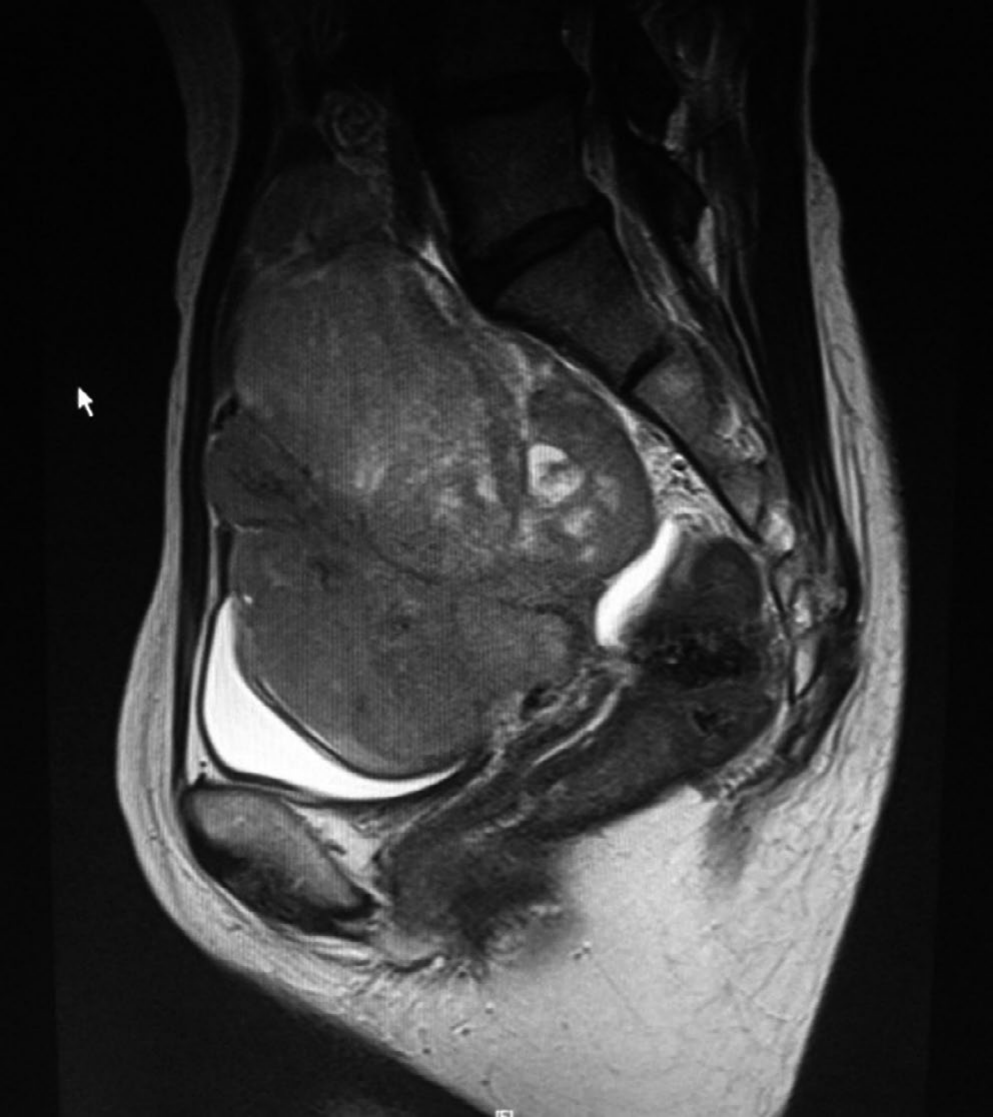
Solid, enhancing right ovarian mass revealed on magnetic resonance imaging

**FIGURE 2:**
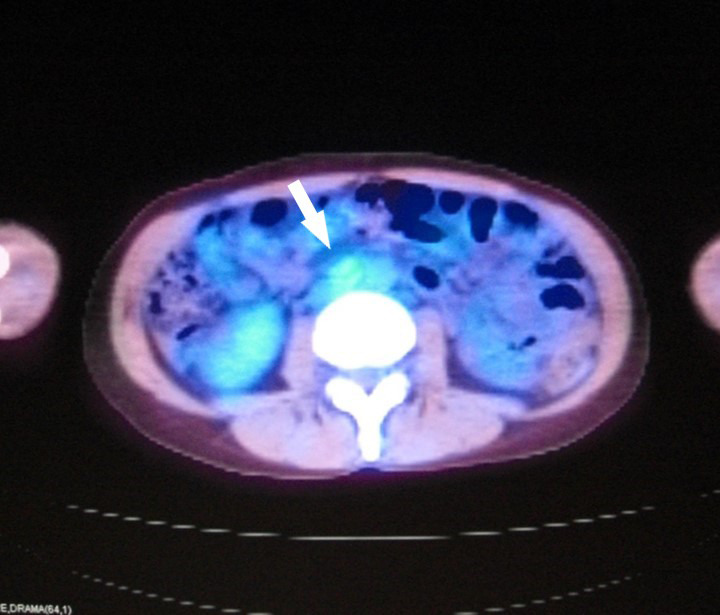
Swollen lymph nodes in the abdominal para‐aorta revealed on positron‐emission tomography and computed tomography (PET/CT: white arrow)

**FIGURE 3:**
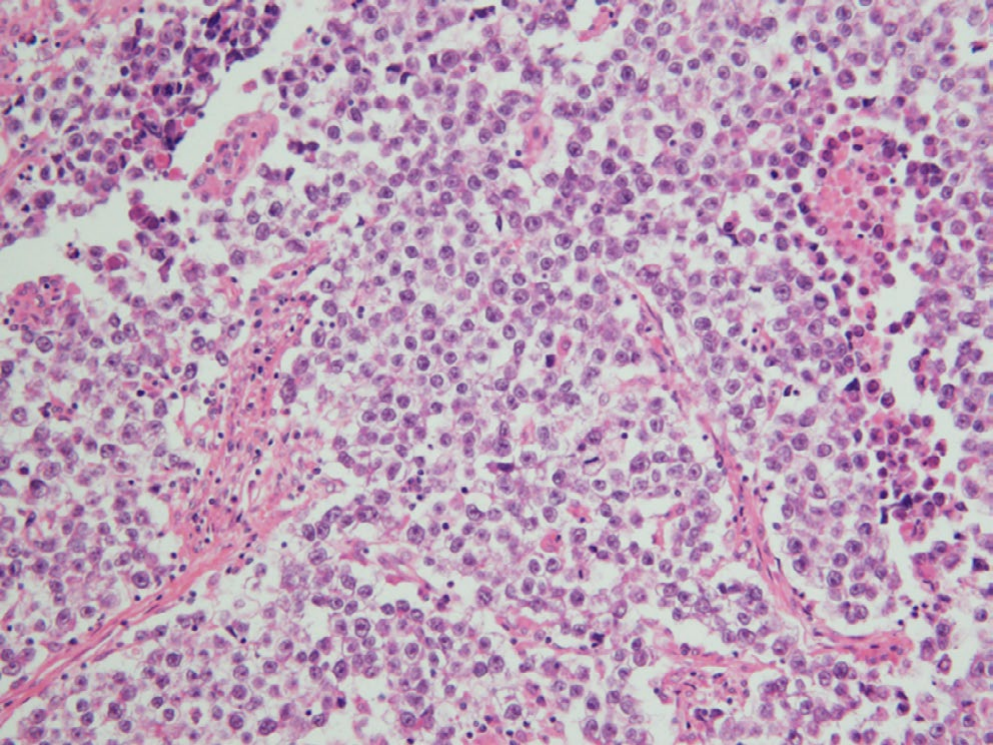
Pathological findings of the ovary: small lymphocytes mixed around the sheets of tumor cells

In the current case, we suspected dysgerminoma preoperatively. Therefore, lymphadenectomy may have been avoided because dysgerminomas usually have excellent prognosis after a simple salpingo‐oophorectomy especially in cases of unilateral tumor without capsular invasion or spread due to their excellent response to chemotherapy.^1^ PAN metastasis in dysgerminoma may be uncommon but occasionally seen. Some case reports have revealed that inadequate initial surgery might lead to under‐staging of presumed FIGO IA malignant dysgerminoma.^2^ Therefore, surgical treatment with complete surgical staging as performed in epithelial ovarian cancers may be needed for dysgerminomas, while fertility‐sparing surgery may be allowed only for young women with limited tumor spread following detailed examination.

## CONFLICT OF INTEREST

All authors declare no conflict of interest relevant to this article.

## AUTHOR CONTRIBUTIONS

RK (primary author) analyzed the data and wrote the manuscript. SS involved formulating the concept of the study, analyzed the data, and drafted the manuscript.

## ETHICAL APPROVAL

Prior to the case study, written informed consent was obtained from the patient. This case report was approved and licensed by the Ethics Committee of the Nippon Medical School.

## CONSENT

Written informed consent was obtained from the patient.

## Data Availability

The data that support the findings of this study are available on request from the corresponding author. The data are not publicly available due to privacy or ethical restrictions.
